# Supplementary material for: Use of Biomarkers in Ongoing Research Protocols on Alzheimer’s Disease
Source: J Pers Med. 2020 Jul 24;10(3):68. doi: 10.3390/jpm10030068 (PMC7564515; doi:10.3390/jpm10030068)
Supplement: Supplementary file 1 [file jpm-10-00068-s001.pdf]

Table S1. Characteristics of the selected protocols registered on the clinicltrials.gov database.

NCT Code	Intervention (s)	Phase	Enrollment
NCT01224106	Drug: Gantenerumab丨Drug: Placebo	Phase 3	799
NCT01300728	Drug: NewGam 10% IVIG丨Other: Placebo	Phase 2	52
NCT01703117	Drug: Riluzole丨Drug: Placebo	Phase 2	42
NCT01760005	Drug: Gantenerumab丨Drug: Solanezumab丨Drug: Matching Placebo (Gantenerumab)丨Drug: Matching Placebo (Solanezumab)	Phase 2丨Phase 3	490
NCT01767311	Drug: BAN2401 2.5 mg/kg丨Drug: BAN2401 5.0 mg/kg丨Drug: BAN2401 10 mg/kg丨Drug: Placebo	Phase 2	856
NCT01811381	Drug: Curcumin丨Behavioral: Aerobic yoga丨Behavioral: Non aerobic yoga丨Dietary Supplement: Placebo	Phase 2	80
NCT01843075	Drug: Liraglutide丨Drug: Placebo	Phase 2	204
NCT01998841	Drug: Crenezumab丨Drug: Placebo	Phase 2	252
NCT02008357	Drug: Placebo丨Drug: Solanezumab	Phase 3	1150
NCT02033941	Drug: Meganatural-Az Grapeseed Extract丨Drug: Placebo	Phase 2	20
NCT02051608	Drug: Gantenerumab丨Drug: Placebo	Phase 3	389
NCT02054208	Biological: Human umbilical cord blood derived mesenchymal stem cells丨Other: Normal saline 2 mL	Phase 1丨Phase 2	45
NCT02085265	Drug: Perindopril丨Drug: Telmisartan	Phase 2	150
NCT02292238	Drug: Benfotiamine	Phase 2	76
NCT02547818	Drug: ALZT-OP1a丨Drug: ALZT-OP1b丨Other: Placebo ALZT-OP1a丨Other: Placebo ALZT-OP1b	Phase 3	620
NCT02600130	Biological: Longeveron Mesenchymal Stem Cells丨Biological: Placebo	Phase 1	33
NCT02646982	Drug: Placebo丨Drug: Candesartan	Phase 2	72
NCT02726906	Behavioral: Moderate-aerobic walking丨Radiation: Positron Emission Tomography (PET) scan丨Behavioral: Healthy living education丨Device: Physical activity monitor	Phase 2	100
NCT02833792	Drug: Human Mesenchymal Stem Cells and Lactated Riunger's Solution丨Other: Placebo	Phase 2	40
NCT02880956	Drug: ABBV-8E12丨Drug: Placebo for ABBV-8E12	Phase 2	453
NCT02899091	Biological: CB-AC-02丨Biological: Placebo	Phase 1丨Phase 2	24
NCT02913664	Drug: Angiotensin II receptor blocker (ARB, losartan) and calcium channel blocker (CCB, amlodipine)丨Behavioral: Aerobic Exercise Training丨Other: Usual Care丨Behavioral: Stretching Exercise丨Drug: Atorvastatin	Phase 2丨Phase 3	513
NCT02925650	Drug: Posiphen丨Drug: Placebo	Phase 1丨Phase 2	24
NCT02931136	Drug: Huperzine A丨Drug: Placebo	Phase 4	300
NCT02947893	Drug: Placebo Capsule(s) Once a Day by Mouth丨Drug: Nilotinib Capsule(s) Once a Day by Mouth	Phase 2	42
NCT02956486	Drug: Elenbecestat (E2609)丨Drug: Placebo	Phase 3	1181
NCT03036280	Drug: Elenbecestat (E2609)丨Drug: Placebo	Phase 3	1018
NCT03061474	Drug: Nicotinamide丨Drug: Placebo Comparator	Phase 2	48
NCT03069014	Drug: 400 mg LM11A-31-BHS丨Drug: 800 mg LM11A-31-BHS丨Drug: Placebos	Phase 1丨Phase 2	242
NCT03090516	Drug: Ginkgo biloba dispersible tablets丨Drug: Donepezil丨Drug: Ginkgo biloba dispersible tablets and Donepezil	Phase 2丨Phase 3	240
NCT03094546	Dietary Supplement: Polyamine丨Dietary Supplement: Placebo	Phase 2	100
NCT03131453	Drug: CNP520 50 mg丨Drug: CNP520 15 mg丨Other: Placebo to CNP520	Phase 2丨Phase 3	1147
NCT03172117	Biological: human umbilical cord blood derived mesenchymal stem cells丨Other: Normal saline 2 mL	Phase 1丨Phase 2	45
NCT03185208	Drug: Lithium Carbonate丨Drug: Placebo oral capsule	Phase 4	80
NCT03186989	Drug: IONIS MAPTRx丨Other: Placebo	Phase 1丨Phase 2	46
NCT03234686	Drug: Deferiprone 600 mg delayed release tablets丨Drug: Placebo Oral Tablet	Phase 2	171
NCT03274817	Drug: Escitalopram Pill丨Drug: Venlafaxine Pill丨Drug: Placebo Oral Tablet	Phase 1	31
NCT03277573	Drug: Salsalate丨Drug: Placebo	Phase 1	40
NCT03282916	Drug: Valacyclovir丨Drug: Placebo	Phase 2	130
NCT03289143	Drug: Semorinemab丨Drug: Placebo丨Drug: [18F] GTP1	Phase 2	457
NCT03361410	Dietary Supplement: Grape Powder丨Dietary Supplement: Placebo Powder	Phase 2	32
NCT03367403	Drug: LY3002813丨Drug: Placebo	Phase 2	266
NCT03417986	Drug: TEP	Phase 2	100
NCT03435861	Drug: VX-745丨Drug: Placebo	Phase 2	40
NCT03441516	Drug: Alfoatirin¬Æ Table + Aripezil¬Æ Tab.丨Drug: Aripezil¬Æ Tab.	Phase 4	79
NCT03443973	Drug: Gantenerumab丨Drug: Placebo	Phase 3	1016
NCT03444870	Drug: Gantenerumab丨Drug: Placebo	Phase 3	1016
NCT03446001	Drug: TRx0237 16 mg/day丨Drug: Placebo丨Drug: TRx0237 8 mg/day	Phase 3	450
NCT03461276	Biological: ABvac40丨Biological: Placebo	Phase 2	120
NCT03486938	Drug: Placebo Oral Tablet丨Drug: AGB101 220 mg tablet	Phase 3	830
NCT03493282	Drug: Active Treatment- CT1812 100 mg丨Drug: Placebo丨Drug: Active Treatment- CT1812 300 mg	Phase 1丨Phase 2	23
NCT03507790	Drug: CT1812丨Drug: Placebo	Phase 2	120
NCT03518073	Drug: LY3303560丨Drug: Placebo	Phase 2	285
NCT03522129	Drug: CT1812丨Drug: Placebo	Phase 1	18
NCT03533257	Drug: AMX0035丨Drug: Placebo	Phase 2	100

NCT03634007	Biological: AAVrh.10hPOE2 vector	Phase 1	15
NCT03649724	Drug: Placebo | Drug: Eligard 22.5 Mg Suspension for Injection	Phase 2	180
NCT03712787	Drug: ABBV-8E12	Phase 2	360
NCT03748303	Drug: Allopregnanolone	Phase 1	12
NCT03752294	Drug: Dabigatran | Drug: Placebo—Cap	Phase 1	40
NCT03790709	Drug: High dose ANAVEX2-73 | Drug: Mid dose ANAVEX2-73 | Drug: Placebo oral capsule	Phase 2 | Phase 3	450
NCT03790982	Drug: Placebo of AD-35 60 mg/AD-35 30 mg | Drug: Placebo of AD-35 60 mg/AD-35 60 mg | Drug: AD-35 30 mg + Placebo of AD-35 30 mg | Drug: AD-35 60 mg	Phase 2	240
NCT03801642	Drug: Dapagliflozin | Other: Placebo	Phase 1 | Phase 2	48
NCT03823404	Drug: COR388 capsule | Drug: Placebo capsule	Phase 2 | Phase 3	573
NCT03828747	Drug: Semorinemab | Drug: Placebo | Drug: [18F] GTP1	Phase 2	260
NCT03865017	Biological: Regulatory T cells | Other: Saline	Phase 1 | Phase 2	20
NCT03867253	Drug: ORY-2001 Low dose | Drug: ORY-2001 High dose | Drug: Placebo	Phase 2	33
NCT03875638	Drug: Levetiracetam | Drug: Placebo oral capsule	Phase 2	85
NCT03880240	Device: Transcranial Alternating Current Stimulation (tACS) | Other: Sham Transcranial Alternating Current Stimulation	Phase 1 | Phase 2	55
NCT03887455	Drug: BAN2401 | Drug: Placebo	Phase 3	1566
NCT03919162	Drug: PQ912 | Other: Placebo	Phase 2	462
NCT03943264	Drug: XPro1595	Phase 1	18
NCT03977584	Drug: Crenezumab | Drug: Placebo | Other: [^18F] GTP1	Phase 2	150
NCT03980730	Drug: Azeliragon | Drug: Placebo	Phase 2 | Phase 3	300
NCT03991988	Drug: Montelukast | Drug: Placebo oral tablet	Phase 2	150
NCT04032626	Drug: Lenalidomide 10 mg | Drug: Placebos	Phase 2	30
NCT04044131	Drug: Metabolic Cofactor Supplementation | Drug: Sorbitol	Phase 2	120
NCT04052737	Drug: Normal Saline along with standard treatment | Drug: PMZ-1620 (sovateltide) along with standard treatment	Phase 2	80
NCT04070378	Drug: Daratumumab Injection	Phase 2	15
NCT04079803	Drug: Placebo oral tablet | Drug: PTI-125 50 mg tablet | Drug: PTI-125 100 mg oral tablet	Phase 2	60
NCT04149860	Drug: Lu AF87908 | Drug: Placebo	Phase 1	96
NCT04187547	Drug: Tricaprilin | Drug: Placebo	Phase 3	300
NCT04191486	Drug: T-817MA | Drug: Placebo	Phase 2	200
NCT04205539	Drug: Dexmedetomidine	Phase 1	50
NCT04228666	Drug: HB-adMSCs	Phase 1 | Phase 2	24
NCT04241068	Drug: Aducanumab	Phase 3	2400
NCT04263519	Drug: Tacrolimus	Phase 2	12
NCT04311515	Radiation: Tau Positron emission tomography (PET) | Radiation: Fluorodeoxyglucose (FDG) Positron emission tomography (PET) | Diagnostic Test: Cerebrospinal fluid (CSF) Biomarkers | Diagnostic Test: Blood Biomarkers | Behavioral: Rating Scales | Drug: PU-AD | Drug: Placebo	Phase 2	150
NCT04314934	Drug: ANAVEX2-73	Phase 2 | Phase 3	450
NCT04339413	Drug: Gantenerumab	Phase 3	173
NCT04374253	Drug: Gantenerumab	Phase 3	2032
NCT04381468	Drug: Pepinemab | Drug: Placebo	Phase 1 | Phase 2	60
